# A General Model for Multilocus Epistatic Interactions in Case-Control Studies

**DOI:** 10.1371/journal.pone.0011384

**Published:** 2010-08-18

**Authors:** Zhong Wang, Tian Liu, Zhenwu Lin, John Hegarty, Walter A. Koltun, Rongling Wu

**Affiliations:** 1 Center for Statistical Genetics, Pennsylvania State University, Hershey, Pennsylvania, United States of America; 2 Pennsylvania State Cancer Institute, Pennsylvania State University, Hershey, Pennsylvania, United States of America; 3 Human Genetics Group, Genome Institute of Singapore, Singapore, Singapore; 4 Department of Surgery, Pennsylvania State University, Hershey, Pennsylvania, United States of America; Institute for Medical Biomathematics, Israel

## Abstract

**Background:**

Epistasis, i.e., the interaction of alleles at different loci, is thought to play a central role in the formation and progression of complex diseases. The complexity of disease expression should arise from a complex network of epistatic interactions involving multiple genes.

**Methodology:**

We develop a general model for testing high-order epistatic interactions for a complex disease in a case-control study. We incorporate the quantitative genetic theory of high-order epistasis into the setting of cases and controls sampled from a natural population. The new model allows the identification and testing of epistasis and its various genetic components.

**Conclusions:**

Simulation studies were used to examine the power and false positive rates of the model under different sampling strategies. The model was used to detect epistasis in a case-control study of inflammatory bowel disease, in which five SNPs at a candidate gene were typed, leading to the identification of a significant three-locus epistasis.

## Introduction

The complexity of biological systems arises from the highly interactive relationships of their components [1], [2]. Thus, it is likely that the metabolic pathways for a phenotypic trait or disease involve multiple interacting gene products and regulatory loci that could generate a complex network of genetic actions and interactions [3], [4]. Current genome-wide linkage or association studies have been able to detect genetic actions of individual genes involved in the phenotypic diversity of a complex trait [5]–[12]. Given its ubiquitousness in controlling complex traits and diseases, epistasis resulting from interactions between alleles at different genes has now received increasing attention in genetic studies [13], [14]. However, many of these studies focus on the identification of low-order pairwise epistasis, leaving epistatic interactions of high orders, their frequency and impact on genetic variation, unexplored.

More recently, Stich et al. [15] developed a linkage mapping approach to uncover three-way interactions among different quantitative trait loci (QTLs) using a mating design. Beerenwinkel et al. [16] proposed a mathematical approach for describing multi-way genetic interactions and employing it to study the genetic structure of fitness landscapes for *Escherichia coli*. Based on the analysis of pathway fragments, Imielinski and Belta [17] used a genome-scale knockout design to detect high-order epistatic relationships between components of large metabolic networks. Hansen and Wagner [18] showed that higher-order genetic interactions are potentially important if the total genomic mutation rate is large and the interaction density among loci is not too low. With the widespread availability of high-throughpout genotyping technology, there is a pressing need to estimate higher-order epistasis involving any number of genes and assess the role of epistasis in the creation and maintenance of genetic variation for complex traits.

The motivation of this study is to develop a general model for estimating epistasis of any order with multilocus single nucleotide polymorphism (SNP) data in case-control studies. In particular, the model allows the estimation and testing of high-order epistasis. Because of its easy sample collection, a population-based case-control design has been widely used in candidate gene or genome-wide association studies [19]–[21]. By comparing genotype frequencies for a gene in unrelated individuals with the disease and healthy controls, this design has power to test the significance of the association between the gene and disease. However, only a few studies used a case-control design to characterize epistasis [19] and, also, the epistasis they defined on the basis of logistic regression models presents a computational complexity. The new model described in this article has, for the first time, embedded quantitative genetic principles into a chi-square test framework, allowing the dissection of overall multilocus genetic effects into various components including epistatic interactions of high orders. The model was validated through simulation studies and a real data analysis.

### Large Quantitative Genetic Models for Epistasis

Epistasis was originally defined as the expression of an allele at one locus masked by an allele at another locus [22]. This concept was then explained in a statistical manner by Fisher [23] as the deviation of genetic action from additivity in a linear model. Fisher's definition allows epistasis to be quantified in different forms based on its biological meaning determined by Bateson [22]. For a two-locus epistasis, all possible forms of epistasis include the interactions between additive effects at the two loci, additive effect at the first locus and dominant effect at the second locus, dominant effect at the first locus and additive effect at the second locus, and dominant effects at the two loci. Each of these epistatic forms contributes differently to the overall genetic value of a two-locus genotype. We used Mather and Jinks' formulation [24] to partition a genotypic value into its different components including epistasis.

### Two-locus Epistasis

Suppose there are two loci, **A** with two alleles 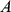 and 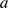 and **B** with two alleles 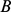 and 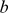, which form nine two-locus genotypes. Let 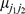 denote the genetic value of an arbitrary genotype 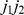 (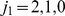 for genotypes 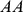, 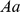, and 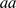; 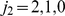 for genotypes 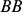, 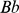, and 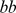, respectively). We dissect 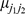 into different components as table 1.

**Table 1:** The genetic effect components of two-locus genotypes.

		Component								
Genotype	Value	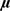	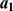	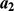	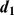	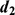	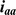	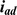	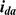	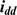
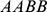	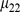	+	+	+			+			
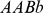	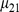	+	+			+		+		
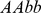	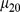	+	+	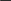			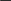			
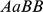	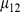	+		+	+				+	
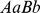	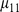	+			+	+				+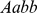	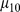	+		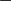	+				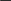	
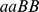	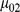	+	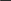	+			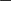			
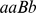	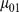	+	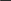			+		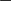		
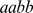	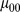	+	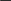	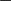			+			

Where 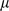 is the overall mean, 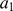 and 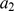 are the additive effect at genes **A** and **B**, 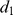 and 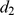 are the dominant effect at genes **A** and **B**, respectively, and 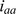, 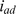, 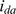, and 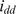 are the additive 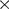 additive, additive 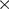 dominant, dominant 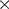 additive, and dominant 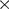 dominant epistatic interactions between the two genes, respectively.

The dissection of genotypic values is expressed, in matrix form, as
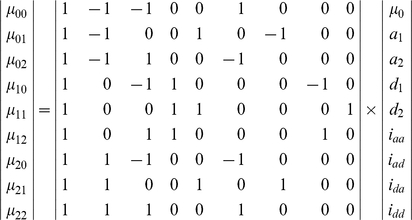
(1)


The genetic effect parameters can be solved using
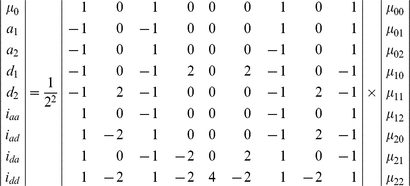
(2)


### Three-locus Epistasis

Adding a locus, **C** with two alleles 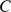 and 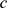, to the two-gene model generates 27 three-locus genotypes, expressed as 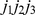 (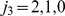 for genotypes 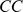, 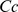, and 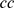, respectively). A three-genotypic value (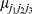) is dissected into the following components:

1. the overall mean 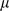;
2. the main genetic effects including the three additive effects (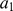, 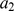, and 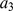) at genes **A**, **B**, and **C**, and the three dominant effects (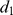, 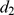, and 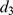) at genes **A**, **B**, and **C**;
3. the two-way interaction effects including the additive 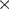 additive (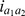), additive 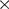 dominant (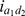), dominant 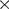 additive (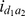), and dominant 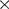 dominant (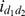) epistasis between genes **A** and **B**, the additive 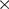 additive (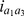), additive 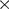 dominant (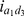), dominant 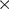 additive (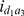), and dominant 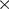 dominant (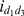) epistasis between genes **A** and **C**, and additive 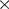 additive (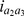), additive 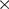 dominant (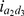), dominant 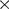 additive (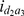), and dominant 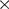 dominant (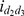) epistasis between genes **B** and **C**;
4. the three-way interaction effects including the additive 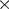 additive 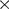 additive (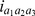), additive 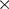 additive 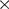 dominant (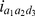), additive 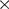 dominant 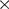 additive (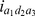), dominant 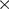 additive 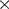 additive (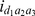), additive 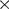 dominant 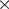 dominant (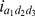), dominant 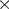 additive 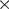 dominant (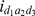), dominant 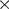 additive 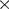 dominant (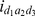), and dominant 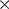 dominant 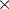 dominant (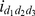) epistasis among genes **A**, **B**, and **C**.

Mather and Jinks' theory is used to formulate the relationships between genotypic values and genetic effects, expressed as
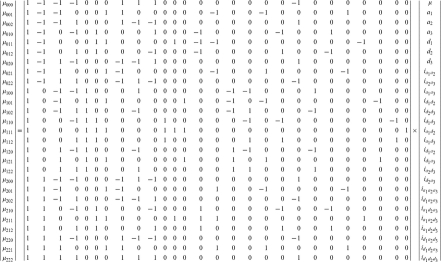
(3)


The genetic effect parameters are then solved from the genotypic values:
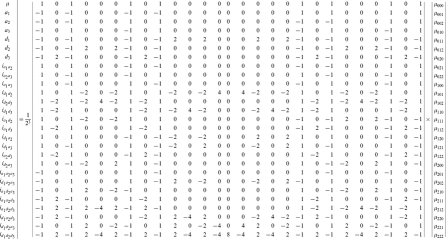
(4)


### N-locus Epistasis

We propose a general model for describing genetic components for a genotype composed of any number of loci. Consider 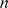 loci which form 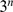 genotypes. The value of a 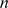-locus genotype is composed of the overall mean, the additive and dominant effects for each locus, and epistasis of different kinds and orders among these loci. Let the space of the genetic effects at individual loci be defined as 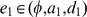 for gene 1, 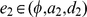 for gene 2, …, 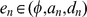 for gene 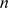. Thus, we can define all possible genetic effects (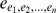) as

- If 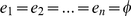, then 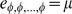;
- If 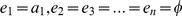, then 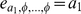;
- …;
- If 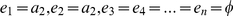, then 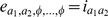;
- …;
- If 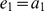, 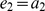, …,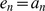, then 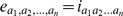.
- …;
- If 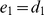, 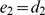, …,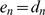, then 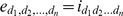.

By letting 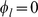, 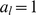 and 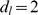 (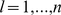), we express the value of a general multi-locus genotype as
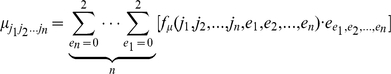
(5)where
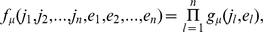
(6)with
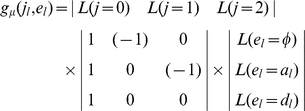
(7)

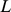
 is a logical judgment function that can return 1 if the condition is true otherwise return 0.

The genetic effect parameters can be estimated by solving the linear equations using
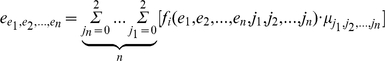
(8)where
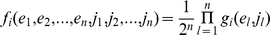
(9)with
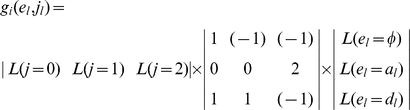
(10)


Equation (8) gives a general form for main and interaction genetic effects among an arbitrary number of loci. Mathematical algorithms for solving epistatic equations are given in Text S1.

### Testing Epistasis

Based on the definitions, we now provide a procedure for testing epistasis of different kinds and orders with multilocus genetic data. Consider a case-control study in which 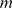 cases (there is a disease) and 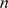 controls (there is no disease) are selected randomly from a natural population. Case and control groups are matched for demographical factors such as age, race, gender, life style, and body mass. All subjects from the case and control groups are genotyped genome-wide or for particular chromosomal regions of interest, depending on the purpose of the study. Let 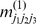 and 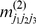 denote the observations of a general genotype 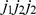 (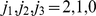) derived from three markers **A**, **B**, and **C**. Based on Mather and Jinks' partition of genotypic values [24], we calculate genetic effect parameters from genotypic values using equation (4). For both cases and controls, the genotypic values used to calculate each effect parameter are dissolved into two groups, plus and minus, which forms a 2 (cases and control)
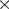
2 (plus and minus) contingency table. For example, the contingency table for testing the additive
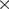
additive
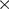
additive epistatic effect is expressed as table 2.

**Table 2:** The 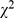 test statistics for the additive
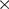
additive
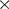
additive epistatic effect.

	Plus	Minus
Cases	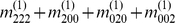	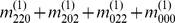
Controls	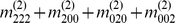	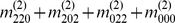

From the table, the 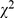 test statistic is calculated and compared with the critical threshold with one degree of freedom. We proved that the test statistics under the null hypothesis calculated from the above contingency table follows a 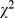 distribution with less than one degree of freedom [25].

The contingency tables for testing the other parameters can be made similarly. For a particular group 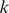 (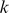 = 1 for cases, 2 for controls), the genotypic values used to calculate the three-way epistatic effect parameters are tabulated as table 3.

**Table 3:** The 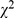 test statistics for the three-way epistatic effect parameters.

Parameter	Plus	Minus
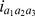	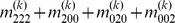	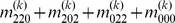
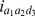	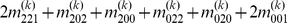	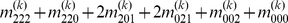
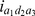	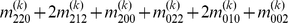	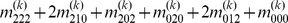
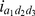	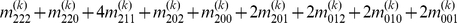	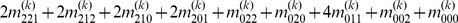
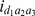	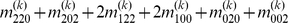	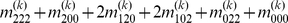
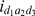	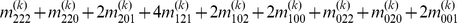	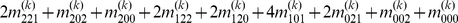
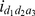	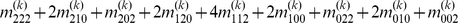	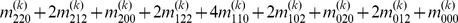
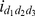	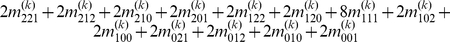	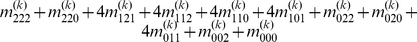

The thresholds for testing each of these three-locus epistases are derived, which are 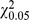 = 3.84, 3.20, 3.20, 3.20, 2.60, 2.60, 2.60, and 2.14, respectively. The genotypic values used to calculate the two-way epistatic effect parameters are tabulated as table 4.

**Table 4:** The 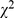 test statistics for the two-way epistatic effect parameters.

Parameter	Plus	Minus
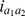	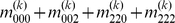	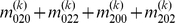
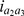	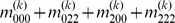	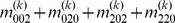
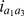	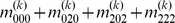	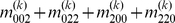
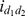	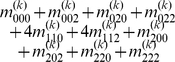	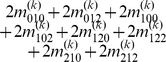
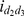	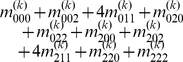	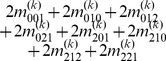
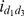	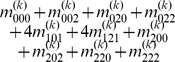	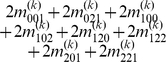
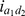	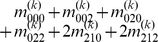	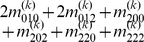
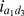	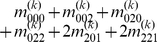	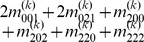
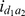	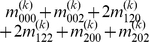	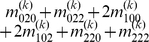
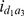	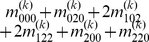	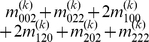
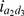	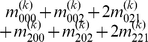	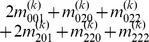
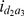	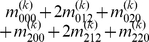	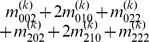

The thresholds for testing each of these two-locus epistases are derived, which are 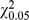 = 3.84, 3.84, 3.84, 2.50, 2.50, 2.50, 3.20, 3.20, 3.20, 3.20, 3.20 and 3.20, respectively. The genotypic values used to calculate the main genetic effect parameters are tabulated as table 5.

**Table 5:** The 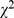 test statistics for the main epistatic effect parameters.

Parameter	Plus	Minus
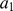	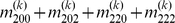	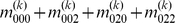
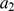	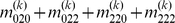	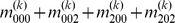
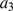	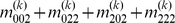	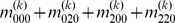
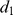	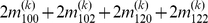	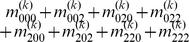
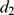	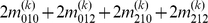	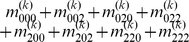
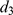	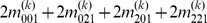	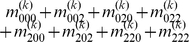

The thresholds for testing each of these two-locus epistases are derived, which are 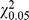 = 3.84, 3.84, 3.84, 2.60, 2.60 and 2.60, respectively. For an arbitrary number of markers, the genotypic values used to calculate the main and epistatic (of different orders) genetic effect parameters can be similarly divided into plus and minus groups, from which the 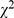 test statistics are calculated.

## Results

The model was used to analyze a case-control study aimed to detect genetic variants for inflammatory bowel disease (IBD) with candidate gene approaches [26]. As a member of the membrane associated guanylate kinase family, TDiscs large homolog (DLG5) plays a central role in maintaining cell junctions and cell shape and in clustering channel proteins at the cell surface [27]. Five single nucleotide polymorphisms (SNPs), Arg30Gln, Glu514Gln, Pro979Leu, Gly1066Gly, and Pro1371Gln, genotyped at DLG5 for both cases and controls are hoped to be associated with IBD. The cases include 115 sporadic IBD patients, aged from 22 to 66 years old, from the Milton S Hershey Medical Center, whereas the controls are 172 unrelated healthy individuals, aged from 15 to 81 years, from the Milton S Hershey Medical Center and Philadelphia gift of Life Donor Program. All the human tissues used for pathological studies and genetic analysis were approved by the Human Subjects Protection Offices of The Pennsylvania State University College of Medicine, and were undertaken with the understanding and written consent of each subject.

Because of a modest sample size used, our analysis will focus on a three-SNP analysis, although the model can deal with any number of SNPs. None of the five SNPs displays an additive genetic effect, but Arg30Gln, Pro979Leu, and Gly1066Gly were each found to trigger a significant dominant effect on the disease (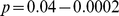) (table 6). There are 10 possible pairs for the five SNPs, with each pair subject to a two-locus epistatic analysis. The number and distribution of two-locus epistasis are given in table 7. It is interesting to see that significant two-locus epistasis was observed only between Arg30Gln and other SNPs including Pro979Leu with a significant main dominant effect and two non-significant SNPs (Glu514Gln and Pro1371Gln). The form of significant epistasis is limited to the interactions between the dominant effect at Arg30Gln and the additive/dominant effects at the other SNPs.

**Table 6:** The 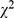 test statistics calculated to test the additive and dominant effects at each SNP genotyped from DLG5.

SNP	Additive	Dominant
	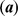	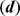
Arg30Gln	1.196	**14.316**
		(0.00015)
Glu514Gln	0	0.355
Pro979Leu	0	**6.095**
		(0.0136)
Gly1066Gly	0.718	**4.297**
		(0.0382)
Pro1371Gln	0	1.933

The 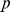-values for those significant effects (in boldface) are given in parentheses.

**Table 7:** The 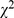 test statistics calculated to test the two-SNP epistasis between each pair of SNPs genotyped from DLG5.

SNP Pair	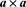	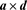	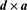	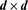
Arg30Gln 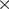 Glu514Gln	0.113	0.112	**3.292**	**2.909**
			(0.040)	(0.020)
Arg30Gln 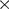 Pro979Leu	0.118	1.085	**3.958**	**2.405**
			(0.025)	(0.040)
Arg30Gln 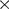 Gly1066Gly	0.005	1.393	1.453	1.741
Arg30Gln 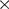 Pro1371Gln	0.107	0.097	**3.184**	**2.740**
			(0.050)	(0.027)
Glu514Gln 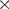 Pro979Leu	0	1.211	0.314	0.340
Glu514Gln 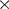 Gly1066Gly	0.222	1.160	0.545	1.205
Glu514Gln 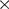 Pro1371Gln	0	0.500	0.107	0.567
Pro979Leu 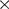 Gly1066Gly	0.261	0.920	0.001	0.607
Pro979Leu 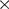 Pro1371Gln	0	0.401	1.434	0.045
Gly1066Gly 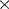 Pro1371Gln	0.290	1.584	1.543	1.907

The 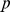-values for those significant effects (in boldface) are given in parentheses.

The five SNPs produce 10 three-locus combinations which were analyzed by a three-locus epistasis model. Each combination has eight forms of three-SNP epistasis. Table 8 lists the test statistics for all possible combinations and forms of epistasis, with significant epistasis highlighted in boldface. The interactions among the additive effects at any three of the five SNPs were not significant; the same was also observed for the three-way dominant interactions. The significant three-locus epistasis must include both the additive and dominant effect at three SNPs. In general, Arg30Gln have more significant three-locus interactions and display higher three-locus significance level than the other SNPs. Arg30Ln, Glu514Gln, and Pro979Leu produce the most numerous forms of epistasis (3), followed by the combinations of Arg30Ln, Gly1066Gly, and Pro1371Gln (2), Glu514Gln, Gly1066Gly, and Pro1371Gln (2), Arg30Ln, Glu514, and Pro1371Gln (1), Arg30Ln, Pro979Leu, and Pro1371Gln (1), Pro979Leu, Gly1066Gly, and Pro1371Gln (1). The three SNPs with significant main effects (Arg30Ln, Pro979Leu, and Pro1371Gln) do not produce a significant three-locus epistatic interaction. The two SNPs displaying non-significant main effects (Glu514Gln and Pro1371Gln) could generate significant three-locus interactions with SNPs Arg30Ln and Gly1066Gly but not with Pro979Leu (table 8).

**Table 8:** The 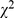 test statistics calculated to test the three-SNP epistasis between each pair of SNPs genotyped from DLG5.

SNP Triplet	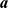 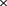 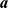 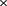 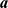	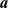 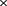 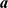 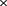 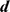	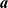 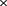 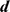 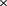 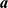	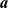 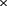 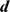 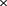 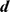	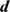 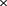 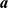 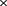 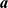	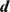 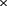 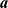 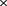 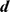	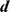 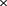 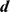 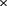 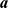	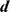 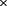 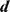 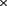 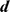
Arg30Ln 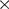 Glu514Gln 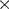 Pro979Leu	0.465	1.274	0.008	0.202	**7.437**	**2.780**	**3.291**	1.533
					(0.0025)	(0.045)	(0.025)	
Arg30Ln 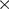 Glu514Gln 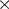 Gly1066Gly	0.010	3.038	0.054	1.499	2.469	0.340	0.815	0.134
Arg30Ln 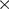 Glu514Gln 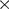 Pro1371Gln	0.426	0.390	0.027	0.183	**5.818**	2.040	2.329	1.011
					(0.008)			
Arg30Ln 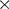 Pro979Leu 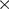 Gly1066Gly	0.002	2.460	0.576	0.772	3.140	0.250	1.151	0.057
Arg30Ln 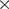 Pro979Leu 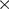 Pro1371Gln	0.448	0.315	1.601	0.014	**7.061**	2.411	2.438	1.127
					(0.0035)			
Arg30Ln 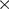 Gly1066Gly 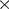 Pro1371Gln	0.005	1.880	**4.250**	**2.618**	3.076	1.652	0.814	0.644
			(0.020)	(0.050)				
Glu514Gln 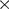 Pro979Leu 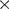 Gly1066Gly	1.101	2.096	0.046	0.908	1.447	1.158	0.120	0.774
Glu514Gln 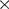 Pro979Leu 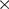 Pro1371Gln	0	0.858	2.262	0.009	0.687	0.672	0.262	0.015
Glu514Gln 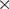 Gly1066Gly 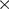 Pro1371Gln	1.191	**3.457**	**3.324**	1.994	1.180	1.832	1.716	1.817
		(0.040)	(0.045)					
Pro979Leu 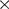 Gly1066Gly 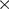 Pro1371Gln	1.322	**3.298**	2.727	1.756	0.100	0.436	1.082	1.255
		(0.050)						

The 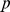-values for those significant effects (in boldface) are given in parentheses.

After significant high-order epistasis is detected, the next step is to make a biological interpretation of such epistasis. To interpret it, we will use the dominant (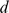)
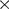
additive (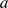)
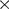
additive (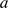) epistasis among Arg30Ln, Glu514Gln, and Pro979Leu as an example. Table 9 gives the structure of genetic effects for each three-locus genotypic value in terms of the additive, dominant, and epistatic effects of different orders. The 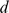 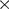 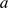 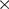 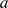 epistasis only contributes to the genotypic value of 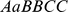, 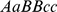, 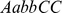, and 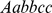 (table 9). For each of these four genotypes, their values are partitioned into different effect components for both cases and controls (table 10). As can be seen, the 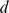 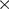 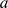 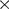 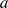 epistasis increases, by 9 cases, the incidence of IBD for those with genotype 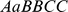 or 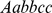, but decreases the IBD incidence of those carrying genotype 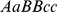 or 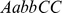 with the same extent.

**Table 9:** Genetic effect components of different three-locus genotypic values.

Genotype	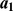	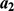	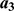	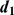	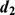	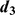	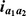	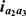	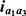	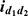	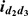	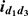	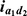	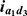	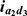	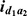	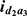	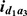	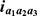	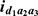	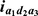	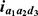	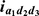	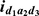	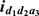	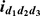
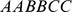	+	+	+				+	+	+										+							
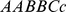	+	+				+	+							+	+							+				
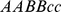	+	+	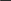				+	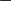	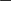										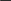							
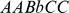	+		+		+				+				+				+				+					
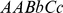	+				+	+					+		+	+									+			
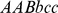	+		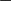		+				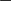				+				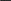				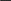					
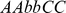	+	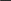	+				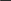	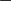	+										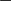							
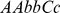	+	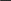				+	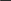							+	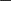							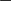				
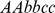	+	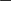	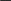				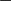	+	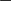										+							
***AaBBCC***		**+**	**+**	**+**				**+**								**+**		**+**		**+**						
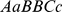		+		+		+						+			+	+							+			
***AaBBcc***		**+**	**−**	**+**				**−**								**+**		**−**		**−**						
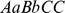			+	+	+					+							+	+							+	
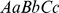					+	+				+	+	+														+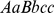			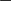	+	+					+							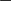	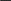							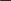	
***AabbCC***		**−**	**+**	**+**				**−**								**−**		**+**		**−**						
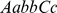		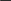		+		+						+			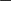	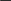								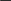		
***Aabbcc***		**−**	**−**	**+**				**+**								**−**		**−**		**+**						
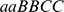	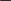	+	+				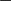	+	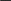											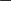						
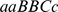	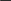	+				+	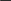							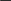	+							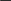				
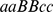	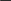	+	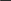				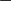	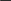	+										+							
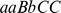	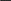		+		+				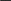				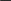				+				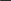					
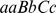	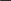				+	+					+		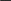	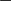									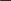			
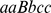	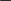		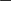		+				+				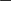				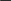				+					
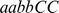	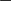	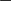	+				+	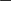	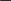										+							
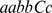	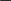	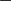				+	+							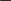	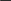							+				
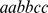	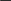	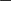	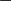				+	+	+										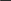							

The genotypic value containing the dominant 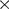 additive 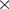 additive epistasis are in boldface.

**Table 10:** The genetic effect components of four particular genotypes, 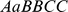, 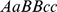, 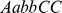, and 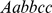 at three SNPs, Arg30Ln, Glu514Gln, and Pro979Leu, which contain the dominant
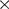
additive
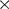
additive three-locus epistasis.

Genotype		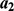	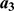	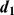	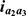	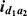	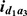	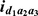
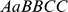	Cases	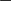 16	16	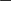 11	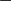 16	11	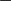 11	11
	Controls	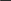 10	10	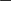 2	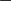 10	2	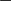 2	2
	Difference	( 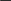 6)	(6)	( 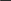 9)	( 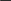 6)	(9)	( 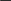 9)	(9)
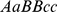	Cases	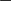 16	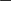 16	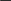 11	16	11	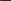 11	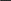 11
	Controls	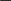 10	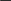 10	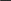 2	10	2	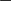 2	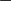 2
	Difference	( 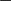 6)	( 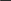 6)	( 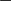 9)	(6)	(9)	( 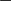 9)	( 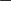 9)
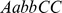	Cases	16	16	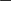 11	16	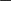 11	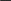 11	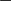 11
	Controls	10	10	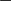 2	10	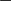 2	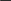 2	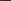 2
	Difference	(6)	(6)	( 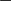 9)	(6)	( 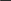 9)	( 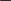 9)	( 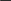 9)
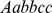	Cases	16	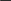 16	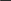 11	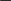 16	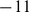	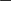 11	11
	Controls	10	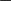 10	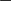 2	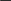 10	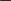 2	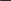 2	2
	Difference	(6)	( 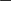 6)	( 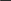 9)	( 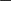 6)	( 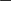 9)	( 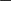 9)	(9)

### Computer Simulation

Simulation studies were undertaken to examine the statistical behavior of the new model. We will focus on the investigation of the power and false positive rates (FDR) for the detection of three-locus epistasis. Three different simulation schemes will be used with varying numbers of cases vs. controls, 200 vs. 200, 400 vs. 400, and 1000 vs. 1000. The eight possible forms of three-locus epistasis can be sorted into four presentative ones, (1) additive
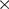
additive
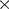
additive (no dominant effect), (2) additive
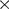
additive
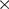
dominant, additive
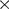
dominant
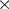
additive, and dominant
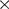
additive
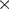
additive (no one dominant effect), (3) additive
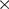
dominant
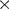
dominant, dominant
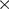
additive
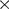
dominant, and dominant
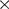
dominant
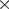
additive (two dominant effects), and (4) dominant
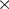
dominant
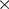
dominant (three dominant effects).

For a real data set, different SNPs may be associated or independent of each other. We will investigate how SNP-SNP associations affect the behavior of the new model. In one data set, three SNPs with the same allele frequency were simulated with pair-wise and three-locus linkage disequilibria. Among the three SNPs, only additive
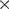
additive
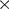
additive, additive
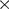
additive
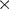
dominant, additive
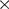
dominant
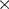
dominant, and dominant
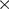
dominant
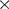
dominant were assumed to exist. This can be done by simulating a contingency table with constraints 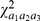 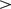 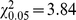, 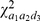 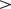 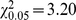, 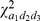 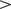 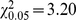, 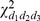 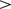 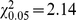 and the test statistics for the other effects 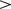 the corresponding thresholds. The same parameters, except that there is no linkage disequilibrium, were used to simulate the second data set containing three SNPs.

Table 11, table 12, and table 13 give the power and false positive error rates (FPR) of the three-locus interaction detection by the new epistatic models. The power to detect the three-locus epistasis increase remarkably with sample size in a case-control study. With sample sizes of 200 vs. 200, there is power of about 0.51–0.61, with the additive
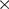
additive
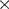
additive epistasis detected most easily, followed by the additive
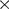
additive
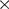
dominant epistasis, the additive
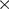
dominant
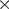
dominant epistasis, and the dominant
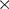
dominant
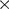
dominant epistasis. When sample sizes increase to 400 vs. 400, the power for the three-locus epistasis detection will surpass three quarters. If sample sizes 1000 vs. 1000 are used, the power reaches 0.99 or more. In general, whether the SNPs are associated or independent does not affect the power substantially, although in some cases the power is higher for associated SNPs than independent SNPs.

**Table 11:** Power and false positive rates (FPR) for the detection of three-locus epistasis among associated and independent SNPs for 200 cases and 200 controls.

	Associated		Independent	
Epistasis	Power	FPR	Power	FPR
Additive 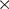 additive 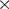 additive	61.2	4.9	51.8	4.0
Additive 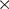 additive 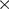 dominant	56.7	4.8	45.2	5.3
Additive 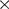 dominant 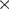 dominant	49.3	5.1	48.4	4.7
Dominant 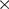 dominant 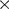 dominant	51.0	6.0	56.1	6.8

**Table 12:** Power and false positive rates (FPR) for the detection of three-locus epistasis among associated and independent SNPs for 400 cases and 400 controls.

	Associated		Independent	
Epistasis	Power	FPR	Power	FPR
Additive 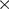 additive 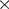 additive	85.0	5.8	79.6	5.4
Additive 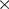 additive 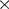 dominant	84.2	4.5	78.6	6.0
Additive 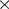 dominant 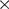 dominant	77.6	5.6	76.8	7.5
Dominant 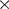 dominant 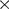 dominant	79.8	7.5	86.6	6.0

**Table 13:** Power and false positive rates (FPR) for the detection of three-locus epistasis among associated and independent SNPs for 1000 cases and 1000 controls.

	Associated		Independent	
Epistasis	Power	FPR	Power	FPR
Additive 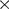 additive 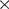 additive	99.8	5.9	99.1	5.6
Additive 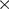 additive 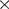 dominant	99.6	4.1	99.3	6.3
Additive 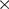 dominant 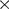 dominant	99.2	5.8	98.0	5.9
Dominant 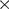 dominant 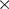 dominant	98.6	8.7	99.7	6.6

The power displays a small FPR (tables 11, 12, and 13). Even if small sample sizes 200 vs. 200 are used, there is still a small chance that the model provides a false positive result for the three-locus epistasis detection. The FPR was found to be consistent, regardless of sample sizes and the degree of SNP-SNP associations.

## Discussion

The phenotypic variation of a trait or disease is highly complex given its polygenic inheritance and environmental influence. Most original quantitative genetic models generally assume that allelic effects are additive, with the size linearly proportional to the number of alleles. These models are modified by considering that there are genetic interactions between different alleles at the same locus (dominance). It is now recognized that the interactions between different loci (epistasis) within gene networks may play an important role [14], [15]. More recent evidence shows that high-order epistasis among more than two genes may form a crucial component in genetic interaction networks [9], [11], [17], [18]. In fact, quantitative genetic analyses have detected high-order epistatic effects in plants. For example, high-order epistasis could be correlated with the aggressiveness of the isolate of *Phytophthora capsici* through influencing double crosses among different loci at meiosis [28]. Wu [29] used a mating design with clonal replicates to identify the significant contribution of high-order epistasis to genetic variation in stem wood growth traits in poplars.

An increasing availability of high-throughput SNP data has led to the development of various statistical approaches for effectively analyzing epistasis among multiple polymorphisms, including logistic regression, multifactor dimensionality reduction (MDR), Bayesian analysis, and machine learning [15], [21], [30]–[32]. In this article, we developed a general model for detecting the episatsis of any order in case-control genetic association studies by integrating traditional quantitative genetic principles. Despite the existence, high-order epistasis may be obscured by metabolic network redundancy [17]. The integration of quantitative genetic principles makes our approach capable to identify high-order epistatic interactions with genetic relevance. The model was tested by simulation studies. It displays adequate power for the detection of high-order epistasis with a modest sample size; for example, 400 cases vs. 400 controls. When sample sizes of cases and controls increase to 1000 vs. 1000, which is currently not a problem for most genetic association studies, the model has almost full power to detect three-locus epistasis of different forms. Even if a small size of samples (say 200 vs. 200), the new model has a low false positive rate for epistatic detection. The practical application of the model is validated by analyzing a real data set for the genetic study of inflammatory bowel disease. The model detected significant three-locus epistatic interactions among different SNPs genotyped from a candidate gene DLG5 [27].

Our model allows the characterization of epistasis of any order. Its implementation into a practical setting of genome-wide association studies is challenged by an exponentially increasing number of SNP-SNP combinations. To make this tractable, one may incorporate optimization techniques into our model, allowing the selection of the most important combinations. An additional issue is to determine the critical threshold with multiple correlated SNPs in genome-wide association studies. An empirical approach for determining a genome-wide threshold is to employ non-parametric permutation testing (see ref. [21], [30], [33]–[35]). Lastly, the model is developed to detect multilocus epistasis at the SNP level, but given recent discoveries for the importance of haplotypes in trait control [36]–[39], the model should be extended to consider high-order interactions expressed by different haplotypes. In the current model specification, we choose controls that are matched for cases in terms of biological, environmental, or demographical factors. When such matches are not possible, we need to embed these factors as covariates into the model, in which the interactions between genes and these factors can be tested. Third, the model can be extended with multiple diseases to consider the pleiotropic effect of a gene. The results about high-order epistasis detection using the this and extended models could be used for iterative model building and functional annotation of genes. Future applications of these results includes analysis of the metabolic networks of pathogenic organisms and generation of epistatic candidate models for genome-wide association studies.

## Supporting Information

Text S1 Mathematical algorithm.
(0.14 MB PDF)
