# Supplementary material for: A General Model for Multilocus Epistatic Interactions in Case-Control Studies
Source: PLoS One. 2010 Aug 18;5(8):e11384. doi: 10.1371/journal.pone.0011384 (PMC2909900; doi:10.1371/journal.pone.0011384)
Supplement: Text S1 — Mathematical algorithm. (0.14 MB PDF) [file pone.0011384.s001.pdf]

Mathematical Algorithms for Solving Epistatic Equations

Define a vector,

$$P_{1\times n}(x) = [0...1...0], \tag{S1}$$

in which the xth element is 1 and all the others are 0. Let g_μ and g_i denote the vectors for values of different genotypes, respectively, which are expressed as

$$g_\mu(t_x, e_x) = P_{1\times n}(e_x) \times M \times P^T_{1\times n}(t_x) \tag{S2}$$

$$g_i(e_x, t_x) = P_{1\times n}(e_x) \times N \times P^T_{1\times n}(t_x) \tag{S3}$$

where

$$M = \begin{bmatrix} 1 & 1 & 1 \\ -1 & 0 & 1 \\ 0 & - & 0 \end{bmatrix} \tag{S4}$$

$$N = \begin{vmatrix} 1 & 0 & 1 \\ -1 & 0 & 1 \\ -1 & 2 & -1 \end{vmatrix} \tag{S5}$$

Algorithm for Two SNPs

The genotypic values for two SNPs are expressed as

$$U(2) = A(2) \times I(2) \tag{S6}$$

where

$$U(2) = \begin{bmatrix} \mu_{00} \\ \mu_{01} \\ \mu_{02} \\ \mu_{10} \\ \mu_{11} \\ \mu_{12} \\ \mu_{20} \\ \mu_{21} \\ \mu_{22} \end{bmatrix}$$

$$A(2) = \begin{bmatrix} f_\mu(0,0,\phi,\phi) & f_\mu(0,0,\phi,a_2) & f_\mu(0,0,\phi,d_2) & f_\mu(0,0,a_1,\phi) & ... & f_\mu(0,0,d_1,d_2) \\ f_\mu(0,1,\phi,\phi) & f_\mu(0,1,\phi,a_2) & f_\mu(0,1,\phi,d_2) & f_\mu(0,1,a_1,\phi) & ... & f_\mu(0,1,d_1,d_2) \\ f_\mu(0,2,\phi,\phi) & f_\mu(0,2,\phi,a_2) & f_\mu(0,2,\phi,d_2) & f_\mu(0,2,a_1,\phi) & ... & f_\mu(0,2,d_1,d_2) \\ f_\mu(1,0,\phi,\phi) & f_\mu(1,0,\phi,a_2) & f_\mu(1,0,\phi,d_2) & f_\mu(1,0,a_1,\phi) & ... & f_\mu(1,0,d_1,d_2) \\ f_\mu(1,1,\phi,\phi) & f_\mu(1,1,\phi,a_2) & f_\mu(1,1,\phi,d_2) & f_\mu(1,1,a_1,\phi) & ... & f_\mu(1,1,d_1,d_2) \\ f_\mu(1,2,\phi,\phi) & f_\mu(1,2,\phi,a_2) & f_\mu(1,2,\phi,d_2) & f_\mu(1,2,a_1,\phi) & ... & f_\mu(1,2,d_1,d_2) \\ f_\mu(2,0,\phi,\phi) & f_\mu(2,0,\phi,a_2) & f_\mu(2,0,\phi,d_2) & f_\mu(2,0,a_1,\phi) & ... & f_\mu(2,0,d_1,d_2) \\ f_\mu(2,1,\phi,\phi) & f_\mu(2,1,\phi,a_2) & f_\mu(2,1,\phi,d_2) & f_\mu(2,1,a_1,\phi) & ... & f_\mu(2,1,d_1,d_2) \\ f_\mu(2,2,\phi,\phi) & f_\mu(2,2,\phi,a_2) & f_\mu(2,2,\phi,d_2) & f_\mu(2,2,a_1,\phi) & ... & f_\mu(2,2,d_1,d_2) \end{bmatrix}$$

$$I(2) = \begin{bmatrix} \mu_0 \\ a_2 \\ d_2 \\ a_1 \\ i_{a_1a_2} \\ i_{a_1d_2} \\ d_1 \\ i_{d_1a_2} \\ i_{d_1d_2} \end{bmatrix}$$

Specifically, we have

$$A(2) = \begin{vmatrix} 1 & -1 & 0 & -1 & 1 & 0 & 0 & 0 & 0 \\ 1 & 0 & 1 & -1 & 0 & -1 & 0 & 0 & 0 \\ 1 & 1 & 0 & -1 & -1 & 0 & 0 & 0 & 0 \\ 1 & -1 & 0 & 0 & 0 & 0 & 1 & -1 & 0 \\ 1 & 0 & 1 & 0 & 0 & 0 & 1 & 0 & 1 \\ 1 & 1 & 0 & 0 & 0 & 0 & 1 & 1 & 0 \\ 1 & -1 & 0 & 1 & -1 & 0 & 0 & 0 & 0 \\ 1 & 0 & 1 & 1 & 0 & 1 & 0 & 0 & 0 \\ 1 & 1 & 0 & 1 & 1 & 0 & 0 & 0 & 0 \end{vmatrix} \tag{S7}$$

By solving equation (S6), we obtain the solution of $I(2)$:

$$I(2) = B(2) \times U(2), \tag{S8}$$

where

$$B(2) = \begin{vmatrix} f_i(\phi,\phi,0,0) & f_i(\phi,a_2,0,0) & f_i(\phi,d_2,0,0) & f_i(a_1,\phi,0,0) & ... & ... & ... & ... & f_i(d_1,d_2,0,0) \\ f_i(\phi,\phi,0,1) & f_i(\phi,a_2,0,1) & f_i(\phi,d_2,0,1) & f_i(a_1,\phi,0,1) & ... & ... & ... & ... & f_i(d_1,d_2,0,1) \\ f_i(\phi,\phi,0,2) & f_i(\phi,a_2,0,2) & f_i(\phi,d_2,0,2) & f_i(a_1,\phi,0,2) & ... & ... & ... & ... & f_i(d_1,d_2,0,2) \\ f_i(\phi,\phi,1,0) & f_i(\phi,a_2,1,0) & f_i(\phi,d_2,1,0) & f_i(a_1,\phi,1,0) & ... & ... & ... & ... & f_i(d_1,d_2,1,0) \\ f_i(\phi,\phi,1,1) & f_i(\phi,a_2,1,1) & f_i(\phi,d_2,1,1) & f_i(a_1,\phi,1,1) & ... & ... & ... & ... & f_i(d_1,d_2,1,1) \\ f_i(\phi,\phi,1,2) & f_i(\phi,a_2,1,2) & f_i(\phi,d_2,1,2) & f_i(a_1,\phi,1,2) & ... & ... & ... & ... & f_i(d_1,d_2,1,2) \\ f_i(\phi,\phi,2,0) & f_i(\phi,a_2,2,0) & f_i(\phi,d_2,2,0) & f_i(a_1,\phi,2,0) & ... & ... & ... & ... & f_i(d_1,d_2,2,0) \\ f_i(\phi,\phi,2,1) & f_i(\phi,a_2,2,1) & f_i(\phi,d_2,2,1) & f_i(a_1,\phi,2,1) & ... & ... & ... & ... & f_i(d_1,d_2,2,1) \\ f_i(\phi,\phi,2,2) & f_i(\phi,a_2,2,2) & f_i(\phi,d_2,2,2) & f_i(a_1,\phi,2,2) & ... & ... & ... & ... & f_i(d_1,d_2,2,2) \end{vmatrix}$$

Specifically, we have

$$B(2) = \frac{1}{2^2}\begin{vmatrix} 1 & 0 & 1 & 0 & 0 & 0 & 1 & 0 & 1 \\ -1 & 0 & 1 & 0 & 0 & 0 & -1 & 0 & 1 \\ -1 & 2 & -1 & 0 & 0 & 0 & -1 & 2 & -1 \\ -1 & 0 & -1 & 0 & 0 & 0 & 1 & 0 & 1 \\ 1 & 0 & -1 & 0 & 0 & 0 & -1 & 0 & 1 \\ 1 & -2 & 1 & 0 & 0 & 0 & -1 & 2 & -1 \\ -1 & 0 & -1 & 2 & 0 & 2 & -1 & 0 & -1 \\ 1 & 0 & -1 & -2 & 0 & 2 & 1 & 0 & -1 \\ 1 & -2 & 1 & -2 & 4 & -2 & 1 & -2 & 1 \end{vmatrix} \tag{S9}$$

Algorithms for n SNPs

For n SNPs, we have an identity matrix,

$$C(n) = A(n) \times B(n) = E_{3^n} \tag{S10}$$

where

$$A(n) = \begin{bmatrix} f_\mu(0,...,0,\phi,...,\phi) & f_\mu(0,..,0,a_n,...,\phi) & f_\mu(0,..,0,d_n,...,\phi) & ... & f_\mu(0,..,0,d_1,...,d_n) \\ f_\mu(0,..,1,\phi,...,\phi) & f_\mu(0,..,1,a_n,...,\phi) & f_\mu(0,..,1,d_n,...,\phi) & ... & f_\mu(0,..,1,d_1,...,d_n) \\ f_\mu(0,..,2,\phi,...,\phi) & f_\mu(0,..,2,a_n,...,\phi) & f_\mu(0,..,2,d_n,...,\phi) & ... & f_\mu(0,..,2,d_1,...,d_n) \\ ... & ... & ... & ... & ... \\ f_\mu(2,...,2,\phi,...,\phi) & f_\mu(2,...,2,a_n,...,\phi) & f_\mu(2,...,2,d_n,...,\phi) & ... & f_\mu(2,...,2,d_1,...,d_n) \end{bmatrix}$$

$$B(n) = \begin{bmatrix} f_i(\phi,...,\phi,0,...,0) & f_i(a_n,...,\phi,0,..,0) & f_i(d_n,...,\phi,0,...,0) & ... & f_i(d_1,...,d_n,0,...,0) \\ f_i(\phi,...,\phi,0,...,1) & f_i(a_n,...,\phi,0,...,1) & f_i(d_n,...,\phi,0,...,1) & ... & f_i(d_1,...,d_n,0,...,1) \\ f_i(\phi,...,\phi,0,...,2) & f_i(a_n,...,\phi,0,...,2) & f_i(d_n,...,\phi,0,...,2) & ... & f_i(d_1,...,d_n,0,...,2) \\ ... & ... & ... & ... & ... \\ f_i(\phi,...,\phi,2,...,2) & f_i(a_n,...,\phi,2,...,2) & f_i(d_n,...,\phi,2,...,2) & ... & f_i(d_1,...,d_n,2,...,2) \end{bmatrix}$$

In general, we have

$$\begin{aligned} C_n(t_1,t_2,...,t_n,e_1,e_2,...,e_n) &= \sum_{k_n=0}^{2} ... \sum_{k_1=0}^{2} [f_\mu(t_1,t_2,...,t_n,k_1,k_2,...,k_n) \cdot f_i(e_1,e_2,...,e_n,k_1,k_2,...,k_n)] \\ &= \frac{1}{2^n} \sum_{k_n=0}^{2} ... \sum_{k_1=0}^{2} (\prod_{x=1}^{n} g_i(e_x,k_x) \prod_{x=1}^{n} g_\mu(t_x,k_x)) \\ &= \begin{cases} 0 & e_x \neq t_x, \exists x \in (1,2,...,n) \\ 1 & e_x = t_x, \forall x \in (1,2,...,n) \end{cases} \end{aligned}$$

Algorithms for $n+1$ SNPs

For $n+1$ SNPs, we will need to prove

$$C(n+1) = A(n+1) \times B(n+1) \tag{S11}$$

is an identity matrix.

We have

$$\begin{aligned}
&C_{n+1}(t_1, ..., t_n, t_{n+1}, e_1, ..., e_n, e_{n+1}) = \\
&\quad = \sum_{k_{n+1}=0}^{2} ... \sum_{k_1=0}^{2} [f_\mu(t_1, ..., t_n, t_{n+1}, k_{n+1}, k_n, ..., k_1) \cdot f_i(e_{n+1}, e_n, ..., e_1, k_{n+1}, k_n, ..., k_1)] \\
&\quad = \sum_{k_n=0}^{2} ... \sum_{k_1=0}^{2} [f_\mu(t_1, ..., t_n, t_{n+1}, k_0, ..., k_n, 0) \cdot f_i(e_1, ..., e_n, e_{n+1}, k_0, ..., k_n, 0)] \\
&\quad + \sum_{k_n=0}^{2} ... \sum_{k_1=0}^{2} [f_\mu(t_1, ..., t_n, t_{n+1}, k_0, ..., k_n, 1) \cdot f_i(e_1, ..., e_n, e_{n+1}, k_0, ..., k_n, 1)] \\
&\quad + \sum_{k_n=0}^{2} ... \sum_{k_1=0}^{2} [f_\mu(t_1, ..., t_n, t_{n+1}, k_0, ..., k_n, 2) \cdot f_i(e_1, ..., e_n, e_{n+1}, k_0, ..., k_n, 2)] \\
&\quad = \frac{1}{2^n} \sum_{k_n=0}^{2} ... \sum_{k_1=0}^{2} (\prod_{x=1}^{n} g_\mu(k_x, t_x) \prod_{x=0}^{n} g_i(e_x, k_x)) \cdot (\frac{1}{2} g_\mu(e_{n+1}, 0) \times g_i(t_{n+1}, 0)) \\
&\quad + \frac{1}{2^n} \sum_{k_n=0}^{2} ... \sum_{k_1=0}^{2} (\prod_{x=1}^{n} g_\mu(k_x, t_x) \prod_{x=0}^{n} g_i(e_x, k_x)) \cdot (\frac{1}{2} g_\mu(e_{n+1}, 1) \times g_i(t_{n+1}, 1)) \\
&\quad + \frac{1}{2^n} \sum_{k_n=0}^{2} ... \sum_{k_1=0}^{2} (\prod_{x=1}^{n} g_\mu(k_x, t_x) \prod_{x=0}^{n} g_i(e_x, k_x)) \cdot (\frac{1}{2} g_\mu(e_{n+1}, 2) \times g_i(t_{n+1}, 2)) \\
&\quad = \frac{1}{2^n} \sum_{k_n=0}^{2} ... \sum_{k_1=0}^{2} (\prod_{x=1}^{n} g_\mu(k_x, t_x) \prod_{x=0}^{n} g_i(e_x, k_x)) \cdot \frac{1}{2} \cdot \sum_{k_{n+1}=0}^{2} (g_\mu(e_{n+1}, k_{n+1}) \times g_i(t_{n+1}, k_{n+1})) \\
&\quad = \frac{1}{2^n} \sum_{k_n=0}^{2} ... \sum_{k_1=0}^{2} (\prod_{x=1}^{n} g_\mu(k_x, t_x) \prod_{x=0}^{n} g_i(e_x, k_x)) \cdot \frac{1}{2} \cdot \varphi(n+1)
\end{aligned}$$

where

$$\varphi(n+1) = \sum_{k_{n+1}=0}^{2} (P_{1\times n}(k_{n+1}) \times M \times P_{1\times n}^{T}(e_{n+1}) \times P_{1\times n}(t_{n+1}) \times N \times P_{1\times n}^{T}(k_{n+1})) \tag{S12}$$

The values of $\varphi(n+1)$ can be obtained from a simple calculation as

	$t_{n+1}=0$	$t_{n+1}=1$	$t_{n+1}=2$
$e_{n+1}=0$	2	0	0
$e_{n+1}=1$	0	2	0
$e_{n+1}=2$	0	0	2

(S13)

from which, we have

$$C_{n+1}(t_1,...,t_n,t_{n+1},e_1,...,e_n,e_{n+1}) = \begin{cases} 0 & e_x \neq t_x, \exists x \in (1,2,...,n+1) \\ 1 & e_x = t_x, \forall x \in (1,2,...,n+1) \end{cases} \tag{S14}$$

Therefore, $A(n+1) \times B(n+1) = E_{3^{n+1}}$ is proven, i.e. $A(n+1)$ is the inverse matrix for $B(n+1)$. We conclude that f_μ can be solved by f_i for any SNPs.

An Example: Three-SNP Model

Genotypic values of three-SNP genotypes are defined as

Genotype	Partition of Genotypic Values
aabbcc	$\mu_{000} = \mu_0 - a_1 - a_2 - a_3 + i_{a_1a_2} + i_{a_2a_3} + i_{a_1a_3} - i_{a_1a_2a_3}$
aabbCc	$\mu_{001} = \mu_0 - a_1 - a_2 + d_3 + i_{a_1a_2} - i_{a_2d_3} - i_{a_1d_3} + i_{a_1a_2d_3}$
aabbCC	$\mu_{002} = \mu_0 - a_1 - a_2 + a_3 + i_{a_1a_2} - i_{a_2a_3} - i_{a_1a_3} + i_{a_1a_2a_3}$
aaBbcc	$\mu_{010} = \mu_0 - a_1 + d_2 - a_3 - i_{a_1d_2} - i_{d_2a_3} + i_{a_1a_3} + i_{a_1d_2a_3}$
aaBbCc	$\mu_{011} = \mu_0 - a_1 + d_2 + d_3 - i_{a_1d_2} + i_{d_2d_3} - i_{a_1d_3} - i_{a_1d_2d_3}$
aaBbCC	$\mu_{012} = \mu_0 - a_1 + d_2 + a_3 - i_{a_1d_2} + i_{d_2a_3} - i_{a_1a_3} - i_{a_1d_2a_3}$
aaBBcc	$\mu_{020} = \mu_0 - a_1 + a_2 - a_3 - i_{a_1a_2} - i_{a_2a_3} + i_{a_1a_3} + i_{a_1a_2a_3}$
aaBBCc	$\mu_{021} = \mu_0 - a_1 + a_2 + d_3 - i_{a_1a_2} + i_{a_2d_3} - i_{a_1d_3} - i_{a_1a_2d_3}$
aaBBCC	$\mu_{022} = \mu_0 - a_1 + a_2 + a_3 - i_{a_1a_2} + i_{a_2a_3} - i_{a_1a_3} - i_{a_1a_2a_3}$
Aabbcc	$\mu_{100} = \mu_0 + d_1 - a_2 - a_3 - i_{d_1a_2} + i_{a_2a_3} - i_{d_1a_3} + i_{d_1a_2a_3}$
AabbCc	$\mu_{101} = \mu_0 + d_1 - a_2 + d_3 - i_{d_1a_2} - i_{a_2d_3} + i_{d_1d_3} - i_{d_1a_2d_3}$
AabbCC	$\mu_{102} = \mu_0 + d_1 - a_2 + a_3 - i_{d_1a_2} - i_{a_2a_3} + i_{d_1a_3} - i_{d_1a_2a_3}$
AaBbcc	$\mu_{110} = \mu_0 + d_1 + d_2 - a_3 + i_{d_1d_2} - i_{d_2a_3} - i_{d_1a_3} - i_{d_1d_2a_3}$
AaBbCc	$\mu_{111} = \mu_0 + d_1 + d_2 + d_3 + i_{d_1d_2} + i_{d_2d_3} + i_{d_1d_3} + i_{d_1d_2d_3}$
AaBbCC	$\mu_{112} = \mu_0 + d_1 + d_2 + a_3 + i_{d_1d_2} + i_{d_2a_3} + i_{d_1a_3} + i_{d_1d_2a_3}$
AaBBcc	$\mu_{120} = \mu_0 + d_1 + a_2 - a_3 + i_{d_1a_2} - i_{a_2a_3} - i_{d_1a_3} - i_{d_1a_2a_3}$
AaBBCc	$\mu_{121} = \mu_0 + d_1 + a_2 + d_3 + i_{d_1a_2} + i_{a_2d_3} + i_{d_1d_3} + i_{d_1a_2d_3}$
AaBBCC	$\mu_{122} = \mu_0 + d_1 + a_2 + a_3 + i_{d_1a_2} + i_{a_2a_3} + i_{d_1a_3} + i_{d_1a_2a_3}$
AAbbcc	$\mu_{200} = \mu_0 + a_1 - a_2 - a_3 - i_{a_1a_2} + i_{a_2a_3} - i_{a_1a_3} + i_{a_1a_2a_3}$
AAbbCc	$\mu_{201} = \mu_0 + a_1 - a_2 + d_3 - i_{a_1a_2} - i_{a_2d_3} + i_{a_1d_3} - i_{a_1a_2d_3}$
AAbbCC	$\mu_{202} = \mu_0 + a_1 - a_2 + a_3 - i_{a_1a_2} - i_{a_2a_3} + i_{a_1a_3} - i_{a_1a_2a_3}$
AABbcc	$\mu_{210} = \mu_0 + a_1 + d_2 - a_3 + i_{a_1d_2} - i_{d_2a_3} - i_{a_1a_3} - i_{a_1d_2a_3}$
AABbCc	$\mu_{211} = \mu_0 + a_1 + d_2 + d_3 + i_{a_1d_2} + i_{d_2d_3} + i_{a_1d_3} + i_{a_1d_2d_3}$
AABbCC	$\mu_{212} = \mu_0 + a_1 + d_2 + a_3 + i_{a_1d_2} + i_{d_2a_3} + i_{a_1a_3} + i_{a_1d_2a_3}$
AABBcc	$\mu_{220} = \mu_0 + a_1 + a_2 - a_3 + i_{a_1a_2} - i_{a_2a_3} - i_{a_1a_3} - i_{a_1a_2a_3}$
AABBCc	$\mu_{221} = \mu_0 + a_1 + a_2 + d_3 + i_{a_1a_2} + i_{a_2d_3} + i_{a_1d_3} + i_{a_1a_2d_3}$
AABBCC	$\mu_{222} = \mu_0 + a_1 + a_2 + a_3 + i_{a_1a_2} + i_{a_2a_3} + i_{a_1a_3} + i_{a_1a_2a_3}$

They are expressed in matrix form as

The genetic effects can be solved by the following equations:

Genotype	a_1	a_2	a_3	d_1	d_2	d_3	$i_{a_1a_2}$	$i_{a_2a_3}$	$i_{a_1a_3}$	$i_{d_1d_2}$	$i_{d_2d_3}$	$i_{d_1d_3}$	$i_{a_1d_2}$	$i_{a_1d_3}$	$i_{a_2d_3}$	$i_{a_2d_1}$	$i_{a_3d_2}$	$i_{a_3d_1}$	$i_{a_1a_2a_3}$	$i_{d_1a_2a_3}$	$i_{a_1d_2a_3}$	$i_{a_1a_2d_3}$	$i_{a_1d_2d_3}$	$i_{d_1a_2d_1}$	$i_{d_1d_2a_3}$	$i_{d_1d_2d_3}$
AABBCC	+	+	+				+	+	+										+							
AABBCc	+	+				+	+							+	+							+				
AABBcc	+	+	-				+	-	-										-							
AABbCC	+		+		+				+				+				+				+					
AABbCc	+				+	+					+		+	+									+			
AABbcc	+		-		+				-				+				-				-					
AAbbCC	+	-	+				-	-	+										-							
AAbbCc	+	-				+	-							+	-							-				
AAbbcc	+	-	-				-	+	-										+							
AaBBCC		+	+	+				+								+		+		+						
AaBBCc		+		+		+						+			+	+							+			
AaBBcc		+	-	+				-								+		-		-						
AaBbCC			+	+	+					+							+	+							+	
AaBbCc				+	+	+				+	+	+														+
AaBbcc			-	+	+					+							-	-							-	
AabbCC		-	+	+				-								-		+		-						
AabbCc		-		+		+						+			-	-								-		
Aabbcc		-	-	+				+								-		-		+						
aaBBCC	-	+	+				-	+	-										-							
aaBBCc	-	+				+	-							-	+							-				
aaBBcc	-	+	-				-	-	+										+							
aaBbCC	-		+		+				-				-				+				-					
aaBbCc	-				+	+					+		-	-									-			
aaBbcc	-		-		+				+				-				-				+					
aabbCC	-	-	+				+	-	-										+							
aabbCc	-	-				+	+							-	-							+				
aabbcc	-	-	-				+	+	+										-							

	μ_{222}	μ_{221}	μ_{220}	μ_{212}	μ_{211}	μ_{210}	μ_{202}	μ_{201}	μ_{200}	μ_{122}	μ_{121}	μ_{120}	μ_{112}	μ_{111}	μ_{110}	μ_{102}	μ_{101}	μ_{100}	μ_{022}	μ_{021}	μ_{020}	μ_{012}	μ_{011}	μ_{010}	μ_{002}	μ_{001}	μ_{000}
μ	1	0	1	0	0	0	1	0	1	0	0	0	0	0	0	0	0	0	1	0	1	0	0	0	1	0	1
a_1	1	0	1	0	0	0	1	0	1	0	0	0	0	0	0	0	0	0	-1	0	-1	0	0	0	-1	0	-1
a_2	1	0	1	0	0	0	-1	0	-1	0	0	0	0	0	0	0	0	0	1	0	1	0	0	0	-1	0	-1
a_3	1	0	-1	0	0	0	1	0	-1	0	0	0	0	0	0	0	0	0	1	0	-1	0	0	0	1	0	-1
d_1	-1	0	-1	0	0	0	-1	0	-1	2	0	2	0	0	0	2	0	2	-1	0	-1	0	0	0	-1	0	-1
d_2	-1	0	-1	2	0	2	-1	0	-1	0	0	0	0	0	0	0	0	0	-1	0	-1	2	0	2	-1	0	-1
d_3	-1	2	-1	0	0	0	-1	2	-1	0	0	0	0	0	0	0	0	0	-1	2	-1	0	0	0	-1	2	-1
i_{a1a2}	1	0	1	0	0	0	-1	0	-1	0	0	0	0	0	0	0	0	0	-1	0	-1	0	0	0	1	0	1
i_{a2a3}	1	0	-1	0	0	0	-1	0	1	0	0	0	0	0	0	0	0	0	1	0	-1	0	0	0	-1	0	1
i_{a1a3}	1	0	-1	0	0	0	1	0	-1	0	0	0	0	0	0	0	0	0	-1	0	1	0	0	0	-1	0	1
i_{d1d2}	1	0	1	-2	0	-2	1	0	1	-2	0	-2	4	0	4	-2	0	-2	1	0	1	-2	0	-2	1	0	1
i_{d2d3}	1	-2	1	-2	4	-2	1	-2	1	0	0	0	0	0	0	0	0	0	1	-2	1	-2	4	-2	1	-2	1
i_{d1d3}	1	-2	1	0	0	0	1	-2	1	-2	4	-2	0	0	0	-2	4	-2	1	-2	1	0	0	0	1	-2	1
i_{a1d2}	-1	0	-1	2	0	2	-1	0	-1	0	0	0	0	0	0	0	0	0	1	0	1	-2	0	-2	1	0	1
i_{a1d3}	-1	2	-1	0	0	0	-1	2	-1	0	0	0	0	0	0	0	0	0	1	-2	1	0	0	0	1	-2	1
i_{a2d3}	-1	2	-1	0	0	0	1	-2	1	0	0	0	0	0	0	0	0	0	-1	2	-1	0	0	0	1	-2	1
i_{a2d1}	-1	0	-1	0	0	0	1	0	1	2	0	2	0	0	0	-2	0	-2	-1	0	-1	0	0	0	1	0	1
i_{a3d2}	-1	0	1	2	0	-2	-1	0	1	0	0	0	0	0	0	0	0	0	-1	0	1	2	0	-2	-1	0	1
i_{a3d1}	-1	0	1	0	0	0	-1	0	1	2	0	-2	0	0	0	2	0	-2	-1	0	1	0	0	0	-1	0	1
i_{a1a2a3}	1	0	-1	0	0	0	-1	0	1	0	0	0	0	0	0	0	0	0	-1	0	1	0	0	0	1	0	-1
i_{d1a2a3}	-1	0	1	0	0	0	1	0	-1	2	0	-2	0	0	0	-2	0	2	-1	0	1	0	0	0	1	0	-1
i_{a1d2a3}	-1	0	1	2	0	-2	-1	0	1	0	0	0	0	0	0	0	0	0	1	0	-1	-2	0	2	1	0	-1
i_{a1a2d3}	-1	2	-1	0	0	0	1	-2	1	0	0	0	0	0	0	0	0	0	1	-2	1	0	0	0	-1	2	-1
i_{a1d2d3}	1	-2	1	-2	4	-2	1	-2	1	0	0	0	0	0	0	0	0	0	-1	2	-1	2	-4	2	-1	2	-1
i_{d1a2d3}	1	-2	1	0	0	0	-1	2	-1	-2	4	-2	0	0	0	2	-4	2	1	-2	1	0	0	0	-1	2	-1
i_{d1d2a3}	1	0	-1	-2	0	2	1	0	-1	-2	0	2	4	0	-4	-2	0	2	1	0	-1	-2	0	2	1	0	-1
i_{d1d2d3}	-1	2	-1	2	-4	2	-1	2	-1	2	-4	2	-4	1	-4	2	-4	2	-1	2	-1	2	-4	2	-1	2	-1
